# Combination of Feature Selection and Resampling Methods to Predict Preterm Birth Based on Electrohysterographic Signals from Imbalance Data

**DOI:** 10.3390/s22145098

**Published:** 2022-07-07

**Authors:** Félix Nieto-del-Amor, Gema Prats-Boluda, Javier Garcia-Casado, Alba Diaz-Martinez, Vicente Jose Diago-Almela, Rogelio Monfort-Ortiz, Dongmei Hao, Yiyao Ye-Lin

**Affiliations:** 1 Centro de Investigación e Innovación en Bioingeniería, Universitat Politècnica de València, 46022 Valencia, Spain; feniede@ci2b.upv.es (F.N.-d.-A.); jgarciac@ci2b.upv.es (J.G.-C.); adiaz@ci2b.upv.es (A.D.-M.); yiye@ci2b.upv.es (Y.Y.-L.); 2 Servicio de Obstetricia, H.U.P. La Fe, 46026 Valencia, Spain; diago_vicalm@gva.es (V.J.D.-A.); monfort_isaort@gva.es (R.M.-O.); 3 Faculty of Environment and Life, Beijing University of Technology, Beijing International Science and Technology Cooperation Base for Intelligent Physiological Measurement and Clinical Transformation, Beijing 100124, China; haodongmei@bjut.edu.cn

**Keywords:** genetic algorithm, imbalance data learning, electrohysterography, preterm labor prediction, resampling methods, uterine electromyography, machine learning

## Abstract

Due to its high sensitivity, electrohysterography (EHG) has emerged as an alternative technique for predicting preterm labor. The main obstacle in designing preterm labor prediction models is the inherent preterm/term imbalance ratio, which can give rise to relatively low performance. Numerous studies obtained promising preterm labor prediction results using the synthetic minority oversampling technique. However, these studies generally overestimate mathematical models’ real generalization capacity by generating synthetic data before splitting the dataset, leaking information between the training and testing partitions and thus reducing the complexity of the classification task. In this work, we analyzed the effect of combining feature selection and resampling methods to overcome the class imbalance problem for predicting preterm labor by EHG. We assessed undersampling, oversampling, and hybrid methods applied to the training and validation dataset during feature selection by genetic algorithm, and analyzed the resampling effect on training data after obtaining the optimized feature subset. The best strategy consisted of undersampling the majority class of the validation dataset to 1:1 during feature selection, without subsequent resampling of the training data, achieving an AUC of 94.5 ± 4.6%, average precision of 84.5 ± 11.7%, maximum F1-score of 79.6 ± 13.8%, and recall of 89.8 ± 12.1%. Our results outperformed the techniques currently used in clinical practice, suggesting the EHG could be used to predict preterm labor in clinics.

## 1. Introduction

### 1.1. Preterm Labor

The World Health Organization defines preterm labor (prevalent in more than 11% of total births) as labor before 37 completed weeks of gestation [1]. It is the leading cause of death in children, accounting for approximately 35% of newborn deaths and 16% of children under five years of age [2]. In the case of survivors, shorter term consequences involve respiratory difficulties, sepsis, neurological conditions, feeding difficulties, as well as visual and hearing problems [3]. Long-term complications include poorer neurodevelopmental outcomes, higher rates of hospital admissions, as well as behavioral, social–emotional, and learning difficulties in childhood [2]. As the average cost of preterm birth is 5–10 times higher than a term birth, preterm birth also has a significant economic impact on public health systems, the average cost of preterm birth is 5–10 times higher than a term birth [4], with an average cost of 64,815 USD per premature baby [5]. For an extremely preterm baby born before 28 weeks of gestation the average cost per baby amounts to 74,009 USD for the first year of life in Germany [6].

Various methods are currently used to predict preterm labor in clinical practice, including: uterine dynamics monitoring by tocodynamometry, cervix length, Bishop score, and bio-chemical markers [7] such as fetal fibronectin and interleukin 6 [8]. None of these techniques can precisely predict true preterm labor, and their clinical values mainly lies in their negative predictive value thanks to their ability to identify patients who are not at risk of preterm labor [7]. Due to its high sensitivity, electrohysterography (EHG) is emerging as a promising technique to identify the risk of preterm birth [9]. This non-invasive technique records the electrical activity of billions of uterine myometrial cells on the maternal abdominal wall.

### 1.2. Electrohysterography for Preterm Labor Prediction

Previous studies showed that the EHG signal distributes its energy within 0.1–4 Hz and is made up of two components: fast wave low (0.2–0.34 Hz), which has been associated with signal propagation, and fast wave high (0.34–4 Hz), which is related to cell excitability [9,10]. Since EHG mainly distributes its energy below 1 Hz, many authors preferred to analyze the signal within the 0.34–1 Hz range to minimize respiration and cardiac interference [11]. Uterine myometrial cell excitability and bioelectric propagability rise due to progressive formation of gap-junctions, which end up leading to coordinated high-intensity contractions that give rise to labor.

A set of temporal, spectral, and non-linear parameters have been proposed in the literature to characterize these electrophysiological changes. As pregnancy progresses, EHG amplitude increases and is associated with a larger number of uterine cells involved in the contractions [9]. The EHG signal spectral content also shifts towards higher frequencies as delivery approaches, suggesting increased cell excitability [9,12]. Previous studies found increased signal regularity, thus reduced complexity, by analyzing Lempel–Ziv and different entropy measures [10,13,14,15,16,17], although controversial results were obtained due to the limited database with different compositions depending on the inclusion criteria and the analysis bandwidth, among others. Time reversibility and Poincaré plot-derived parameters were also used for characterizing the EHG signal [13,14,18], with an increased signal non-linearity degree and less randomness as pregnancy progresses.

The latest research studies focused on the development of preterm birth prediction systems and have obtained promising results, with an accuracy of more than 90% [11,15,19,20,21]. However, they have not had a significant impact on clinical praxis. Firstly, most preterm labor prediction systems used complex classifiers that involve the non-linear transformation of input features into higher dimension space to better separate the target classes [22]. Obstetricians find the prediction results difficult to interpret and hard to trust, since these algorithms achieve good performance even when the input features are highly overlapped between the target classes [19,23]. In this regard, we have shown the feasibility of predicting preterm labor with the synthetic minority oversampling technique (SMOTE) on a balanced dataset using simple classification algorithms such as the K-nearest-neighbor, logistic regression, and linear discriminant analysis by feature subspace optimization using a genetic algorithm [15,19]. Secondly, due to the highly imbalanced data between the two target classes (11% preterm labor vs. 89% term labor), conventional classification algorithms are often biased towards the majority class and fail to correctly identify the minority class, obtaining a higher misclassification rate of true preterm labor in predicting premature deliveries [21,24,25,26]. This phenomenon is due to the fact that conventional machine learning algorithms are designed to optimize the overall performance (accuracy) instead of considering the predictive capability of each class [27]. The majority class data are relatively excessively distributed than the minority class data, thus invading the minority class area and hindering the correct setting of the decision boundary [25].

### 1.3. Resampling Methods for Imbalance Data Learning

Rebalancing to equal the distribution of data classes is a commonly used strategy to mitigate the above imbalanced learning problems. Most previous studies used SMOTE, which consisted of synthesizing new samples by interpolating the original minority class observations [28], achieving promising results [11,15,19,26,29,30,31,32]. Nevertheless, according to a recent study [26], these works may overestimate preterm labor prediction performance due to their methodological bias. Application of the SMOTE technique prior to data partition would give rise to the data structure correlation between training and test dataset, and tends to overestimate the real generalization capacity of the model [26]. In fact, Vandewiele et al. attempted to reproduce the preterm labor prediction system method of 11 published studies and analyzed the model’s performance difference between applying SMOTE before and after data partition [26]. When balancing data before partition, they obtained an AUC ranging from 85% to 99% which was very close to the reported evaluation metrics. In contrast, when applying SMOTE to training data after partitioning, prediction performance decreased drastically, with an AUC below 65% using the same input features and classification algorithms [26]. Due to the underlying assumption of the homogeneity of the clusters of minority observations, SMOTE can inappropriately alter the class distribution when factors such as disjoint data distributions, noise, and outliers are present [33]. In addition to the SMOTE technique, other resampling methods have also been proposed to mitigate the imbalanced data problem, including undersampling and oversampling/undersampling hybrid methods [21]. Undersampling is a non-heuristic method that consists of removing instances from the majority class to alleviate the skewed class distribution problem. This latter is limited to a moderate or low imbalanced dataset and is not recommended for highly imbalanced datasets because of its high potential of underfitting due to information loss [34]. If the size of the minority class sample is small the classifier performance may be greatly impaired [34]. However, other authors have proposed hybrid oversampling/undersampling methods to reduce the class overlap problem, which usually consists of cleaning the majority class observations in proximity to the minority instances by the undersampling method before or after SMOTE [35,36,37,38].

Studies in different application areas have attempted to determine the optimal resampling method from a database set with variable numbers and/or type characteristics [34,39,40]. Napierala & Stefanowski studied types of minority class distribution in real imbalanced datasets and their influence on learning classifiers [39]. Zhou analyzed the effect of sampling methods on the performance of quantitative bankruptcy prediction models on real highly imbalanced dataset and confirmed that the proper sampling method in developing prediction models mainly depended on the size of the training sample [40]. With hundreds of minority observations in the dataset, the undersampling was superior to the oversampling method in terms of computation time, although SMOTE was found to be a better choice with only a few dozen minority instances. A combination of SMOTE and undersampling could be a good alternative for a large training sample [40]. Loyola-González et al. analyzed the impact of resampling methods for contrast pattern based classifiers on imbalanced databases and provided a guide for the selection of the resampling method regarding the class imbalance ratio [34]. Despite these previous studies, no resampling method always outperforms the others [41]. It is difficult to determine a specific optimal rate of undersampling or oversampling which always leads to better results for a specific application [41].

Other authors have proposed combining feature selection, resampling, and ensemble learning to deal with multiclass imbalanced data learning, and obtained results that outperformed or were comparable to several state-of-the art algorithms [42]. In the classification task, high-dimensional features may lead to overfitting, which can limit the model’s generalization capability [43]. Removing irrelevant features may reduce the noise information in the training space and also model complexity and training time. In imbalanced scenarios, high-dimensionality could have a greater impact; as minority class samples can easily be discarded as noise [42], eliminating irrelevant features may also reduce the risk of treating the minority class as noise. High-dimensionality can even lead to class overlapping, which makes the design of discriminative rules extremely difficult in imbalanced data scenarios [44]. Ramos-Pérez et al. analyzed the combination effects of resampling and feature selection techniques on high-dimensional and low instance imbalanced data, also determining whether resample data should be before or after feature selection [45]. The contribution of feature selection to specific preterm labor prediction from imbalanced data remains unclear.

The aim of this work was to determine the effect of combining feature selection and resampling methods on preterm labor prediction from imbalanced data. We first confirmed that the application of resampling methods before data partition considerably reduced the complexity of the classification task. We showed the feasibility of combining both the feature selection using genetic algorithm and resample methods in the same iterative process to deal with imbalanced data, in contrast to resampling before or after feature selection. Our results suggested that undersampling the validation set turned out to be the best strategy for preterm labor prediction in an imbalanced scenario, achieving a recall ranging from 79.6% to 89.8%, which is considerably higher than the techniques commonly used in clinical practice and also than the unbiased preterm labor prediction performance reported by Vandewiele et al.

## 2. Materials and Methods

### 2.1. Database Description

**Figure 1:**
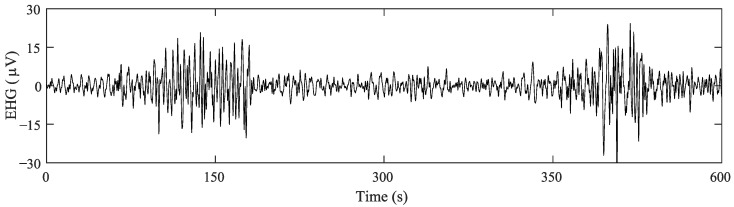
Example of preprocessed EHG signal recorded from women with 30 weeks of gestation who finally delivered at preterm. Two EHG-bursts associated with uterine contraction can be clearly seen (around 150 s and 400 s) with increased amplitude and frequency contents with respect to basal activity when the uterus is at rest.

300 EHG records from “Term-Preterm EHG Database” (TPEHG DB) [10] and 26 EHG records from “The Term-Preterm EHG Dataset with tocogram” (TPEHGT DS) [46] obtained between 22 and 37 weeks of gestation were analyzed in the study. This ensemble database was highly imbalanced in terms of preterm labor: 275 term labor (84%) vs. 51 preterm (16%). Both datasets used the same recording protocol, which consisted of placing four electrodes (E1, E2, E3 and E4) on the abdomen to obtain three bipolar channels (S1, S2 and S3), with a pairwise distance of 7 cm. All the signals were sampled at 20 Hz and then pre-processed by band-pass filtering between 0.1 and 4 Hz using a fifth-order digital zero-phase Butterworth filter (see Figure 1). We also used obstetric data available from both databases, such as maternal age, parity, number of previous abortions, maternal weight and weeks of gestation on recording.

### 2.2. EHG Signal Analysis

As EHG signal recordings may not only contain uterine myoelectrical activity, but also corrupt segments such as motion-artifacts and respiratory interference, EHG records were reviewed by two experts in a double-blind process to remove all the corrupted signal segments. A whole windows analysis with sliding windows of 120 s length and 50% overlap was then performed to characterize the EHG recordings [10,13,14], and proved to be a good trade-off between computational cost and information loss [47]. This type of analysis was able to identify relevant information in the EHG signal without identifying EHG-bursts associated with uterine contractions [47], which could be very challenging in EHG records taken far from delivery. After obtaining all the features of the analysis windows of a whole recording, we computed the median value as the representative data of this process.

We used a widely used set of temporal, spectral, and non-linear parameters for EHG signal characterization. First, we calculated EHG signal peak-to-peak amplitude (App) in the following four bandwidths: 0.1–4 Hz, 0.2–0.34 Hz, 0.34–4 Hz and 0.34–1 Hz. Since EHG spectral content mainly distributed its energy in the 0.2–1 Hz bandwidth, we estimated dominant frequency DF1 in the range 0.2–1 Hz, DF2 in 0.34–1 Hz, normalized sub-band energy (NormEn) (0.2–0.34 Hz, 0.34–0.6 Hz and 0.6–1 Hz) and high (0.34–1 Hz)-to low (0.2–0.34 Hz) frequency energy ratio (H/L ratio). We also calculated mean frequency (MeanF), power spectrum deciles (D1, …, D9), Teager energy and spectral moment ratio (SpMR) in 0.2–1 Hz. Likewise, we computed the following parameters to quantify the non-linear degree, signal complexity and regularity: binary and multistate Lempel–Ziv index (LZBin and LZMulti n = 6), time reversibility (TimeRev), Katz fractal dimension (KFD), Poincaré ellipse metrics (minor axis (SD1), major axis (SD2), square root of variance (SDRR, $\sqrt{{(\mathrm{SD}1}^{2}{+\mathrm{SD}2}^{2})/2}$) and SD1/SD2 ratio), sample entropy (SampEn), fuzzy entropy (FuzEn), spectral entropy (SpEn), dispersion entropy (DispEn), and bubble entropy (BubbEn) [15]. Since non-linear parameters estimated from different bandwidths may contain complementary information for predicting preterm labor [14], we computed the non-linear parameters in the same four bandwidths as the signal amplitude. In total, each record was characterized by a set of 222 EHG features ((4 temporal, 18 spectral, and 52 non-linear parameters per channel)·3 channels = 222) and the 5 obstetric patient data. Table 1 summarizes all the parameters described in this section, which constituted the input features of the preterm labor prediction system.

### 2.3. Classifier Design and Evaluation

Our specific application was first characterized by a total of 227 high-dimensional input features, with few and imbalanced sample data between the target classes (326 EHG records, with an imbalanced ratio of 51/275 preterm/term cases). We used the conventional holdout method (200 partitions) to design and validate the classifier. For each partition, the whole imbalanced database was randomly split into training (80%) and testing (20%), preserving the skewness between the preterm and term classes (preterm/term samples = 51/275). The training partition was then further split into training (64%) and validation datasets (16%). As mentioned above, we attempted to evaluate the effect of combining feature selection and resampling methods for predicting preterm labor in an imbalanced scenario. As there is still no general agreement in the literature as to which strategy with imbalanced data obtains the best performance, we compared the different strategies by balancing training or validation data using the following resampling methods: oversampling (SMOTE, k = 5), undersampling, and over/undersampling hybrid, the preterm/term instance ratio after data balancing being 1:1.We used the neighborhood cleaning rule (NCL) for the undersampling method; this uses Wilson’s edited nearest neighbor rule to remove noise instances, as it identifies the boundary samples to the decision boundary to avoid overfitting [48].

Step 1: effect of resampling strategy for feature selection. We used the genetic algorithm to optimize feature subspace, which has been proven to successfully preserve complementary information for predicting preterm labor in the SMOTE balanced database, while discarding redundant, irrelevant and noise information [15,19]. This algorithm (GA) is an optimization technique, a population-based heuristic search method that simulates the natural evolutionary process. It is an iterative procedure that manipulates a population of chromosomes (solution candidates) to produce a new population through genetic functions such as crossing over and mutation. These algorithms have been shown to be able to escape from local minima to reach global minima in complex functions [49]. We used the same GA configuration parameters as in our previous studies (see Table 2) [15,19].

As for the classification method, in this work we used the simple easily interpreted linear discrimination analysis (LDA) to discriminate the target classes, which has obtained good results for predicting preterm birth in previous works [15,19]. The mathematical formulation of LDA classification methods can be found in previous works [22].

All the chromosomes in the total population were evaluated to determine model goodness by the fitness function, which we defined as the mean F1-score of the 200 validation datasets weighted by the number of features used in each iteration [15,19,49]. This was used in preference to accuracy, since the F1-score is the geometric mean of precision and recall and obtains the correct classification of the preterm observation, without ignoring term observations.

**Figure 2:**
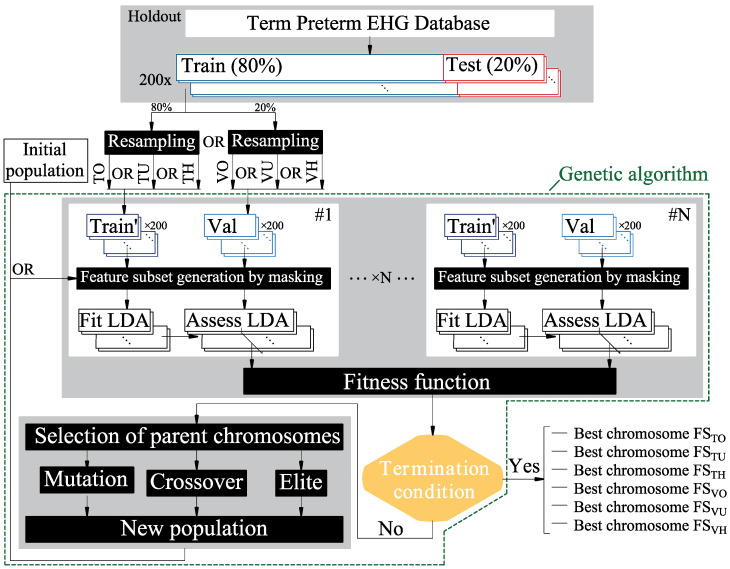
Flowchart to assess the effect of combining feature selection by the genetic algorithm and resampling methods to deal with the imbalanced data problem. The training or validation partitions are resampled by oversampling (TO, VO), undersampling (TU, VU), or applying hybrid methods (TH, VH). The initial population of N chromosomes masks the training and validation partitions. For each chromosome, LDA classifiers are trained and evaluated with the respective validation partitions by its fitness function. A new population of chromosomes is generated from the processes of mutation, crossing over, and selection of the elite chromosomes from the previous iteration until the termination condition was satisfied, obtaining its corresponding best chromosome.

Fitness function = mean{F1-score × (NFeat − NCFeat)}(1)

where NFeat and NCFeat are the number of features in the initial set and the current subset, respectively. The six best chromosomes which optimized feature subsets were thus obtained by considering the following assumptions: resampling the training partition by oversampling (FS_TO_), undersampling (FS_TU_), or under/oversampling hybrid (FS_TH_) method; resampling the validation partition by oversampling (FS_VO_), undersampling (FS_VU_), or under/oversampling (FS_VH_) hybrid method. Figure 2 shows the flowchart that assesses the effect of combining feature selection by the genetic algorithm and the different resampling methods for imbalanced data learning.

**Figure 3:**
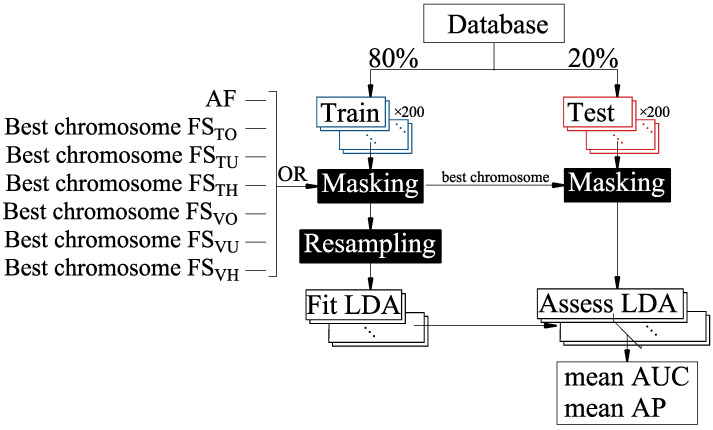
Flow diagram of the training process and evaluation of the prediction models.

Step 2: effect of resampling strategy for training the prediction model. For each optimized feature subset, we further assessed the influence of the different resampling methods (RN, RO, RU and RH, see Table 3) applied to the total of 80% of training dataset (see Figure 3). Each training and test partition was masked by the optimized feature subset FS_TO_, FS_TU_, FS_TH_, FS_VO_, FS_VU_ or FS_VH_ or not (all features, AF). We then trained the LDA classifier using the resampled training partition and evaluated its average performance for the testing dataset, which represents the new incoming data never seen by the model and could be used to determine the real model generalization capability, using two threshold independent metrics to evaluate the model performance: the area under the ROC Curve (AUC) and average precision (AP). This was because the threshold-dependent metrics have been shown to be biased towards the majority class in an imbalanced scenario, whereas AUC and AP avoid this bias [50]. AUC and AP are mathematically formulated in Equations (2) and (3).

(2) $$\mathrm{AUC}=\int_{0}^{1}\mathrm{TPR}(\mathrm{FPR})\;\times \;\mathrm{dFPR}\;$$

(3) $$\mathrm{AP}=\underset{n}{\sum }\left(R_{n}{\;-\;R}_{n-1}\right){\;\times \;P}_{n}$$

where TPR and FPR are true positive rate and false positive rate, and R_n_ and R_n−1_ are the precision and recall at the nth threshold. 

We then analyzed the statistically significant difference between the different model performances to determine the best strategy to achieve the highest average AUC and AP scores ((AUC + AP)/2) for the testing dataset. We first confirmed the normal data distribution (D’Agostino’s k-squared test [51]) for both AUC and AP scores of the 200 partitions for each combination of feature subset and resampling method. Then we assessed the statistically significant difference of the (AUC + AP)/2 between different resampling methods for each feature subset by one-way analysis of variance with repeated measures (RANOVA, α = 0.05) followed by Tukey’s multiple comparison test and evaluated the statistically significant difference of the (AUC + AP)/2 between the different feature subsets for all the resampling methods (RN + RO + RU + RH) by the same statistical method (α = 0.05).

Step 3. Effect of imbalance ratio for feature extraction. We also assessed the influence of the post-resampling preterm/term instance ratio (imbalance ratio) for the best strategy of steps 1 and 2 (resampling methods for feature subset and training of prediction model). The process shown in Figure 2 was again used to obtain nine best chromosomes with an imbalance ratio of from 20 to 100% with a 10% step. We determined the statistically significant differences of the model performances between the different imbalanced ratios using the same statistical method (α = 0.05).

Finally, for the best strategy, i.e., the best (AUC + AP)/2, we determined the threshold-dependent scores of the test partitions for the operative point that maximizes the F1-score and G-mean: F1-score, G-mean, precision, recall, and specificity. Recall metric denotes the true preterm birth predicted by the algorithm with respect to the total of preterm labor women in the testing partition. Precision represents the true preterm birth with respect to the total preterm birth predicted by the algorithm. Specificity refers to the true negative rates over the total negative cases predicted by the algorithm. F1-score is the harmonic average of recall and precision, which is a trade-off between false positives and false negatives. G-mean was defined as the geometric average of recall and specificity [52]. All these metrics were mathematically formulated in the Equations(4)–(8) [53].

(4) $$\mathrm{recall}=\frac{\mathrm{TP}}{\mathrm{TP}+\mathrm{FN}}$$

(5) $$\mathrm{specificity}=\frac{\mathrm{TN}}{\mathrm{FP}+\mathrm{TN}}$$

(6) $$\mathrm{precision}=\frac{\mathrm{TP}}{\mathrm{TP}+\mathrm{FP}}$$

(7) $$F1-\mathrm{score}=\frac{2\;\times \;\mathrm{recall}\;\times \;\mathrm{precision}}{\mathrm{recall}+\mathrm{precision}}$$

(8) $$G-\mathrm{mean}=\sqrt{\mathrm{recall}\;\times \;\mathrm{specificty}}$$

where TP is the true positive, TN is the true negative, FP is the false positive, and FN is the false negative.

## 3. Results

**Figure 4:**
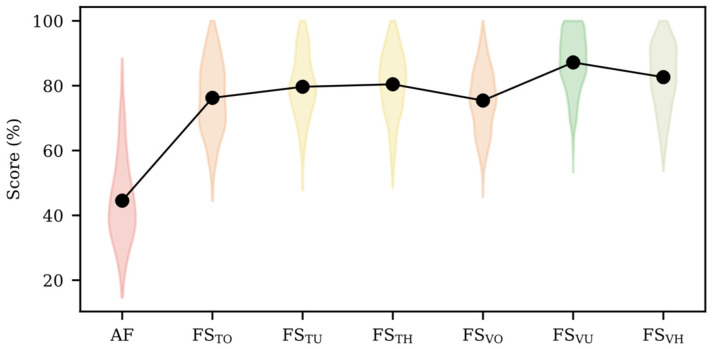
Violin plots represent the distribution of (AUC + AP)/2 for the four resampling methods for each set of input features and average value of (AUC + AP)/2 in black line. Violin color represents homogenous group with similar performance without significant difference (*p* > 0.05).

Table 4 shows the average AUC and AP scores for the testing dataset to predict preterm labor in an imbalanced scenario using each combination of the resampling method for the feature subset and training of the prediction model. Figure 4 shows the violin plot of the score (AUC + AP)/2 for the four resampling methods of each set of input features. The average values of AUC, AP and (AUC + AP)/2 are also shown in this figure. When using all features (AF) for designing the model, SMOTE (RO) did not enhance the prediction capacity of the base classifier with AUC~52% and AP~21%. Both the undersampling (RU) and hybrid (RH) methods performed significantly better, achieving an AUC of ~65% and AP of ~12%. When using FS_TO_, FS_TU_ and FS_TH_ as input features, different resampling methods yield similar performance with no significant difference. For FS_VO_, FS_VU_ and FS_VH_, the no resampling (RN) and oversampling (RO) versions performed significantly better than the undersampling and hybrid versions. When using the optimized feature subset achieved by the genetic algorithm (FS_TO_, FS_TU_, FS_TH_, FS_VO_, FS_VU_ and FS_VH_), none of the resampling methods proposed for the training dataset of the models significantly improved the model performance without additional resampling (RO, RU, RH vs. RN).

The different optimized feature subsets obtained by the genetic algorithm significantly improved the mean score of AUC and AP over AF. Undersampling or hybrid methods during feature selection achieved significantly higher mean AUC and AP scores than those obtained by the oversampling method when used in the training or validation subsets (FS_TU_ ≈ FS_TH_ > FS_TO_, and FS_VU_ ≈ FS_VH_ > FS_VO_). Regarding whether to balance training or validation datasets during feature selection, better performance metrics were obtained for the latter in all cases (except for AP(FS_TO_) vs. AP(FS_VO_)). Undersampling the validation dataset significantly outperformed the rest (FS_VU_ > FS_VH_ > FS_VO_). Our results showed that the best preterm labor prediction strategy in an imbalanced scenario was undersampling the validation dataset for feature selection, with no further resampling method (base-classifier RN of FS_VU_).

**Figure 5:**
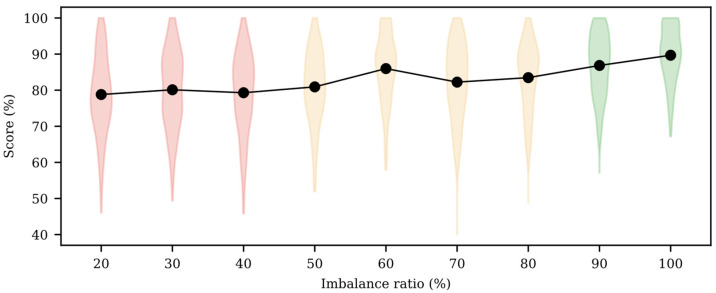
(AUC + AP)/2 distribution of testing partition for optimum feature subset obtained from undersampling validation partition with different imbalance ratios and the average value of (AUC + AP)/2 (black line). Violin colors represent homogenous groups with similar performance and no significant differences (*p* > 0.05).

We also evaluated the effect on the model performance of post-resampling the imbalance ratio of the validation dataset. Table 5 shows AUC and AP values for the optimized features subset achieved using different validation dataset ratios. Besides, Figure 5 shows violin plots of the score (AUC + AP)/2. Ratios of from 20% to 40% performed significantly worse than the other imbalance ratios. The model performance increased from an imbalance ratio of 50%, with the best result achieved when the validation partition was totally balanced. The statistical analysis showed that imbalance ratios of 90% and 100% significantly outperformed those of 50–80%, with no statistically significant differences between them. The number of features included in each best chromosome was 30, 44, 35, 34, 46, 57, 55, 59, and 58 for imbalance ratios of from 20% to 100%, respectively.

**Figure 6:**
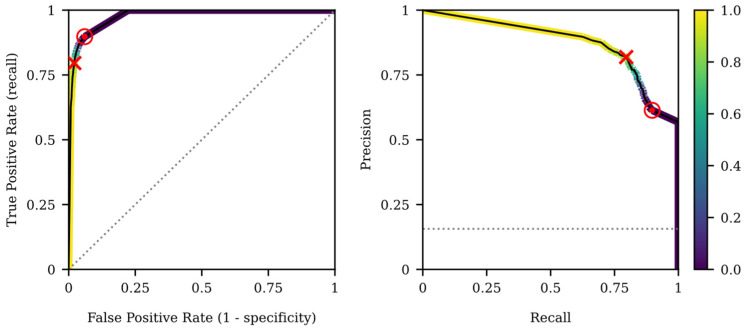
Average ROC curve (left) and precision-recall curve (right) of the testing dataset for the best combination of feature subset and resampling method (FS_VU_, imbalance ratio 100% and no resampling). The red “x” and “⦿” markers show the operative points that maximize the F1-score and G-mean, respectively. The threshold level was shown for each point of the color curves (more blue means closer to 0 and more yellow closer to 1). The dotted lines in the graphs represent the ROC baseline and precision-recall curves (random classifier).

Figure 6 shows the average ROC and precision-recall curve for the best strategy to deal with the imbalanced data problem (FS_VU_, imbalance ratio 100% and no resampling method) for the testing dataset. Table 6 shows the threshold-dependent scores for the test partitions for the operative point that maximizes the F1-score (threshold = 0.85) and G-mean (threshold = 0.01) shown in Figure 6. The maximum F1-score for preterm labor prediction in an imbalanced scenario was 79.6 ± 13.8%, with a recall of 79.6 ± 17.4% and precision of 81.9 ± 14.9% for testing dataset. By maximizing the G-mean we can further improve the recall score to 89.8 ±12.1% with a specificity of 94 ± 5.4%.

## 4. Discussion

### 4.1. Imbalanced Data Learning

This paper describes different resampling methods for dealing with the imbalanced class problem to predict preterm labor from EHG records and obstetrical data and identified their realistic generalization capability for new incoming data. To avoid data structure correlation by oversampling the whole database before data partition, this was carried out before resampling. Regardless of the resampling method, we found that poor results were obtained when using all input features due to high dimensionality, achieving an AUC of less than 65% and AP below 40%. This result may suggest the existence of noise information that could give rise to high data overlapping between the target classes. These results were comparable with those obtained by Vandewiele et al., who obtained an AUC < 65% applying SMOTE after data partition without optimizing the feature subspace [26]. Other authors found that oversampling before data partition significantly reduced the classification task complexity [54], i.e., training and testing data have a similar and correlated data structure, overestimating the model’s generalization capability [26,54]. Indeed, we found the classification task complexity considerably increased with respect to oversampling before data partition. In fact, in the present work the optimum feature set (FS_VU_ and no resampling method) for preterm labor prediction was compounded by 58 features, which were much more than the 12-feature subset using the SMOTE balanced dataset before partition [15].

Regardless of the resampling method applied to the training or validation data, the feature optimization of the subspace by the genetic algorithm may reduce the overlapping data between the target classes and classification task complexity [55,56], thus significantly increasing both AUC and AP. Our results revealed the importance of feature quality in correctly discriminating target classes in an imbalanced data scenario. The optimized feature subset achieved by balancing data using the oversampling method performed worse than the undersampling method. This may be due to the ability of the latter method to remove noisy observations close to the decision boundary, thus increasing the visibility of the minority class and reducing classification task complexity [25,57]. The reduced data overlap enhanced sensitivity, highly desirable in the medical context, while offering good trade-offs between the majority and minority class accuracy rates [57]. By contrast, SMOTE may alter class distribution in the presence of noise and/outlier instances [33], unavoidable in medical data, giving rise to blurring of the decision boundary between the target classes [58]. We also found that the undersampling validation dataset performed significantly better than the balancing training data (FS_TU_ vs. FS_VU_ columns, Table 4), which by the undersampling method eliminate a great deal of information of the majority class for the training model. The total sample size used to design the model was thus too small to statistically represent their population, worsening the quality of the feature subspace and impairing the classifier performance [34]. However, the hybrid resampling method performed significantly better than the oversampling method, being slightly, but significantly, worse than undersampling, suggesting that the latter is the main cause of the relative improvement of the model performance in hybrid implementations.

After obtaining the optimized feature subset after balancing the validation data by the undersampling method, it was no longer necessary to apply the resampling method to the training data. In fact, similar results were obtained for the original data without the resampling and oversampling method. Again, as applying the undersampling method to the training data could even worsen the model performance due to information loss [21,24] (see AP: RN vs. RU, Table 4), there was an insufficient sample size to design a robust preterm labor prediction system. Our results suggest that both the feature selection and resampling methods are effective to solve the classification task in imbalanced scenarios. These results agree with other authors who studied the combined feature selection and resampling method for imbalance data learning and found that in 79% of the study cases, balancing before feature selection improves the results [59]. We also showed the feasibility of combining both the feature selection and resampling methods in the same iterative process to deal with imbalanced data, in contrast to resampling before or after feature selection [59,60]. Balancing validation data to deal with the imbalance data problem was similar to the strategy proposed by Jain et al., who used a weighted sum of recall and specificity as the fitness function [61]. By adding more weight to the recall metric, minority samples became more representative in the fitness function, thus to some extent overcoming the bias of the classifier towards the majority class [61].

Conventional accuracy is known to be unsuitable for evaluating classifier performance in an imbalanced scenario, although in the literature both the F1-score and G-mean have been widely used for this purpose [24,52,62]. Many studies highlight the weakness of the threshold-dependent metric in comparison to threshold-independent metrics such as AUC and AP in imbalanced scenarios [63,64]. Jeni et al. compared a broad range of metrics that included both threshold-dependent metrics (accuracy, F1-score, Cohen’s kappa, and Krippendorf’s alpha) and threshold-independent metrics such as AUC of the ROC curve and precision-recall curve [62]. They found that all other metrics except threshold-independent metrics were attenuated by skewed distributions. Although the area under the ROC is a popular and strong measure to assess the performance of binary classifiers, it has been found that the ROC curve may provide an overly optimistic view when dealing with imbalanced data [27,65]. By contrast, precision-recall curves can be more informative than ROC and have become the basis for assessing performance imbalanced data learning [27,65]. In fact, a very different precision-recall would be obtained for the same ROC in these scenarios (see Table 5). In the present work we used threshold-independent metrics, as suggested by other authors [27,65,66], to avoid a data-skewed bias. Threshold-independent metrics avoid the optimization of the threshold for class assignment and ease the preliminary comparison of different classifier performances. After obtaining the best strategy to achieve the highest AUC and AP mean score, we further determined the threshold-dependent metrics by maximizing both the F1-score and G-mean. By maximizing the latter mean, we considerably increased the recall score by reducing the false negative cases that consisted of true preterm labor patients misclassified as term cases, despite the fact that this necessarily involved less precision [62]. The false negative cases in our application are especially relevant in obstetrics, due to the serious consequences of preterm birth on the newborn’s health.

### 4.2. Preterm Labor Prediction System

Using the optimized feature subset obtained by undersampling the validation dataset, our best results achieved an AUC~94% and AP~84%. Although this result may perform worse than most studies in the literature that attempted to predict preterm birth by balancing the data by SMOTE before data partition [11,15,19,29,32,67], there is no comparison from the methodological point of view. We believe that the generalization capability of the preterm term prediction model in these studies is overestimated, due to the leaked information between the training and testing partitions [26]. Our model outperformed that obtained by Vandewiele et al. who, as in the present work, conducted data partition before the resampling method [26].

The fact that we did not obtain even better results was due to diverse main factors. In addition to a small database with an imbalance problem, the features from the preterm and term classes were highly overlapping, since the EHG data was recorded a considerable time before delivery. Our results agree with other authors who found that the impact of class imbalance on sensitivity greatly depends on the degree of class overlap [25,68], i.e., class imbalance had a greater impact when class overlap was high and seemed insignificant when low. For the case under study, a total of 326 registers were considered when the imbalance ratio was (preterm cases/term cases = 51/275). There is some evidence that overlapping between classes is the main cause of misclassification for this amount of records and imbalance ratio [25]. Other difficult factors, such as small sample size, the presence of disjoint data distribution, outlier and noise observations, and high dimensionality features could be amplified by the data imbalance, making the classification task more challenging [25]. The influence of the data imbalance problem decreases for larger datasets; when the train data is large enough imbalanced distributions do not prevent correct classification, even when the imbalance level is very high [69,70]. There are only a few dozen minority instances in our application that can cause a possible distribution discrepancy between the training, validation, and testing data. Considering that 10% of all births will deliver preterm, an effect size of 0.2, error margin of 5%, and confidence level of 95%, at least 27 preterm women were necessary in the training, validation, and testing data to statistically represent the overall population [71]. This means approximately 810 patients were required to design a robust and generalizable preterm labor prediction system for clinical use. There is currently an urgent requirement for a large database of EHG records to determine its clinical value for predicting preterm labor. In this regard, although there are other publicly available EHG databases, we were unable to join databases from different sources due to the lack of a standardized protocol for data acquisition [21]. In addition, these databases were obtained from women in regular check-ups, which means that some important preterm birth prediction measures, such as cervical length, fetal fibronectin and/or interleukin 6 [11] are missing from their obstetric data [10,46]. Including these additional clinical data in the classifier could therefore further enhance preterm labor prediction performance [13].

### 4.3. Limitations, Future Works and Practical Implications

Our results suggest that the best strategy to mitigate imbalanced data learning in highly overlapping classification tasks with small samples, which is very frequent in the medical data context, is to undersample the validation dataset to 1:1 during feature selection. Despite the promising results, the present work is not exempt from limitations: in addition to the limited sample size, we only tested our method by LDA classification methods in a specific application. This general recommendation should be further corroborated by future studies that seek to deal with imbalanced data learning using other classification methods and/or to be used for other classification tasks.

Future work may be directed toward the use of other strategies to mitigate the imbalanced data problem, such as cost-sensitive or ensemble learning [21], which to date has only been used to predict preterm births from EHG records using balanced data by oversampling before data partition. In spite of the limitations of our study, we believe that the results faithfully represent a realistic generalization capacity for new incoming data, with a recall ranging from 79.6% to 89.8%, which is considerably higher than the techniques commonly used in clinical practice [7,72,73,74]. Our results contribute to more accurate prediction and prevention of preterm labor, which is highly relevant in clinical practice. Accurate prediction of preterm labor would allow screening out almost 75% of false threatened preterm labor cases, with an estimated cost of 20,372 USD/patient [75], which would give rise to substantial savings for public health systems. It would also allow clinicians to provide better and more personalized care to real preterm labor cases, potentially contribute to increasing the survival rate in cases of extreme prematurity by prolonging pregnancy, and reduce long-term morbidity and lifelong disabilities in survivors.

## 5. Conclusions

In the present work we have shown the feasibility of combining different resampling methods in feature selection and training the prediction model during the same iterative process to deal with the imbalanced data problem. We found that overlapping data between the target classes was the main problem in predicting preterm labor and was amplified by the data imbalance scenario. Feature selection by the genetic algorithm and intrinsically balancing the validation partition could significantly reduce data overlap between target classes and improve the model performance. This result highlights the importance of the feature quality for preterm labor prediction. Using the best chromosome by the genetic algorithm, subsequent resampling of the training dataset did not improve decision making, suggesting that the same feature subset was already optimally arranged to avoid information loss and noise between observations.

We also determined that the undersampling method during feature selection outperformed the oversampling method, thanks to its ability to enhance the visibility of the minority class by eliminating noisy observations close to the decision boundary, while undersampling seemed to be the main contribution of the model performance improvement in hybrid implementations. The best strategy to mitigate imbalanced data consisted of undersampling the validation dataset to 1:1 during feature selection, achieving an AUC~94% and AP~84%. The maximum F1-score was around 80%, with a recall of ~80%. By maximizing the G-mean, the best model achieved a recall of ~90%, with an F1-score around 72%. Our results represent a realistic estimation of the EHG technique’s generalization capability for predicting preterm labor and outperform the current techniques used in clinical practice to detect true preterm labor cases, thus constituting a useful tool for clinical use for preterm labor prevention.

## Figures and Tables

**Table 1:** EHG features and obstetrical data included as input data to the classifier to discriminate preterm from term deliveries. The number of features per channel depends on the frequency bandwidths and were computed: 0.1–4 Hz, 0.2–0.34 Hz, 0.34–4 Hz and 0.34–1 Hz for temporal and non-linear features, and 0.2–1 Hz for spectral features. Considering: peak to peak amplitude (APP), dominant frequency (DF1) in the range 0.2–1 Hz, (DF2) in 0.34–1 Hz, normalized sub-band energy (NormEn) (0.2–0.34 Hz, 0.34–0.6 Hz and 0.6–1 Hz) and high (0.34–1 Hz)-to low (0.2–0.34 Hz) frequency energy ratio (H/L ratio), power spectrum deciles (D1, …, D9), spectral moment ratio (SpMR), binary and multistate Lempel–Ziv index (LZBin and LZMulti), time reversibility (TimeRev), Katz fractal dimension (KFD), Poincaré ellipse metrics (minor axis (SD1), major axis (SD2), square root of variance (SDRR, $\sqrt{{(\mathrm{SD}1}^{2}{+\mathrm{SD}2}^{2})/2}$) and SD1/SD2 ratio), sample entropy (SampEn), fuzzy entropy (FuzEn), spectral entropy (SpEn), dispersion entropy (DispEn), and bubble entropy (BubbEn).

EHG Temporal Features	EHG Spectral Features	EHG Non-Linear Features	Obstetrical Data
4 per channel	18 per channel	52 per channel	5
App	MeanF DF1, DF2 NormEn H/L Ratio [D1–D9] Teager Energy SpecMR	LZBin LZMulti (n = 6) TimeRev KFD SD1 SD2 SDRR SD1/SD2 SampEn FuzEn SpEn DispEn BubbEn	Maternal age Parity Abortions Weight Week of gestation at recording time (Wog)

**Table 2:** Configuration parameters used in genetic algorithm.

Parameter	Value	Parameter	Value
Population size	N = 222	Mutation	Uniform
Genome length	N = 222	Mutation Probability	0.01
Number of generations	500	Selection scheme	Tournament of size 2
Crossover	Arithmetic	Elite count	2
Crossover Probability	0.8	Termination condition	No fitness function improvement for 150 consecutive iterations (differential tolerance: 10−6)

**Table 3:** Resampling method for predicting preterm labor.

Approach	Resampling Technique	Abbreviation
No resampling	Not applicable	RN
Oversampling	SMOTE	RO
Undersampling	Neighborhood Cleaning Rule	RU
Hybrid	SMOTE + Neighborhood Cleaning Rule	RH

**Table 4:** AUC and AP scores for the testing datasets for each feature subset and resampling method. Homogenous groups of resampling method with similar performance with no statistically significant differences are shown in different shades of grey. Bottom row shows the average value of the four resampling methods for each feature subset.

**AUC** **(%)**		**AF**	**FS_TO_**	**FS_TU_**	**FS_TH_**	**FS_VO_**	**FS_VU_**	**FS_VH_**
	**RN**	52.1 ± 12.3	86.7 ± 8.2	90.3 ± 6.6	89.9 ± 6.6	88.8 ± 5.5	94.5 ± 4.6	93.5 ± 4.4
	**RO**	52.8 ± 12.2	86.5 ± 8.0	90.6 ± 6.0	90.2 ± 6.4	89 ± 5.8	93.7 ± 4.8	92.5 ± 5.2
	**RU**	65.6 ± 11.7	86.7 ± 8.3	90.9 ± 6.3	89.2 ± 7.2	85.8 ± 6.9	92.9 ± 5.3	91.2 ± 5.5
	**RH**	65.1 ± 12.2	85.9 ± 7.9	91.5 ± 5.3	89.9 ± 6.93	87.4 ± 6.2	92.3 ± 5.7	89.9 ± 6.3
	**mean**	59.2 ± 13.9	86.5 ± 8.1	90.8 ± 6.1	89.9 ± 6.8	88.2 ± 5.9	93.4 ± 5.2	91.8 ± 5.5
**AP** **(%)**		**AF**	**FS_TO_**	**FS_TU_**	**FS_TH_**	**FS_VO_**	**FS_VU_**	**FS_VH_**
	**RN**	22.9 ± 8.9	66.5 ± 16.1	70.3 ± 15.4	70.6 ± 14.5	63.6 ± 14.5	84.8 ± 11.7	77.8 ± 14.4
	**RO**	21.6 ± 7.2	65.3 ± 15.8	67.1 ± 15.6	70.2 ± 15	65.4 ± 14.7	82.9 ± 11.9	75.6 ± 14.3
	**RU**	36.7 ± 15.5	66.7 ± 15.7	69.1 ± 15.5	69.6 ± 15.9	57.4 ± 14.9	78.4 ± 14.1	71.5 ± 15.6
	**RH**	36.7 ± 14.7	64.9 ± 15	67.3 ± 15.0	70.7 ± 14.9	60.6 ± 15.1	77.7 ± 14.4	69.1 ± 15.9
	**mean**	29.9 ± 14.5	66 ± 15.8	68.5 ± 15.3	71.0 ± 15.0	62.7 ± 14.9	81.0 ± 13.4	73.5 ± 15.4

**Table 5:** AUC and AP scores for the testing datasets for optimum feature subset obtained from undersampling validation partition with different imbalance ratios.

**Imbalance Ratio (%)**	**20**	**30**	**40**	**50**	**60**
AUC (%)	86.9 ± 8	88.9 ± 7.4	86.5 ± 8.5	89.2 ± 6.9	90.4 ± 7.2
AP (%)	70.7 ± 15.1	71.3 ± 15.1	72.1 ± 14.6	72.7 ± 14.9	81.6 ± 13
**Imbalance Ratio (%)**	**70**	**80**	**90**	**100**	**-**
AUC (%)	88.5 ± 8.6	91.4 ± 5.7	92.5 ± 5.7	94.5 ± 4.6	-
AP (%)	76 ± 14.2	75.5 ± 14.5	81.2 ± 12.7	84.8 ± 11.7	-

**Table 6:** Threshold-dependent metrics for the best model (FS_VU_, imbalance ratio 100% and no resampling method).

Maximizing Criteria	F1-Score (%)	G-Mean (%)	Precision (%)	Recall (%)	Specificity (%)
F1-score	79.6 ± 13.8	87.7 ± 10.1	81.9 ± 14.9	79.6 ± 17.4	97.9 ± 2.7
G-mean	71.5 ± 17.8	91.6 ± 6.7	61.4 ± 21.5	89.8 ± 12.1	94.0 ± 5.4

## Data Availability

Access and download of the data used are openly available at https://physionet.org, accessed on 29 June 2022. “Term-Preterm EHG Database” (TPEHG DB) is available at https://www.physionet.org/content/tpehgdb/1.0.1/ (accessed on 29 June 2022) and the “The Term-Preterm EHG Dataset with tocogram” is available at https://physionet.org/content/tpehgt/1.0.0/ (accessed on 29 June 2022).

## References

[1] WHO (1977). Recommended definitions, terminology and format for statistical tables related to the perinatal period and use of a new certificate for cause of perinatal deaths. Modifications recommended by FIGO as amended 14 October 1976. Acta Obstet. Gynecol. Scand..

[2] Vogel J.P., Chawanpaiboon S., Moller A.-B., Watananirun K., Bonet M., Lumbiganon P. (2018). The global epidemiology of preterm birth. Best Pract. Res. Clin. Obstet. Gynaecol..

[3] Mandy G.T. (2019). Short-term complications of the preterm infant. UpToDate.

[4] Petrou S., Yiu H.H., Kwon J. (2019). Economic consequences of preterm birth: A systematic review of the recent literature (2009–2017). Arch. Dis. Child..

[5] Waitzman N.J., Jalali A., Grosse S.D. (2021). Preterm birth lifetime costs in the United States in 2016: An update. Semin. Perinatol..

[6] Jacob J., Lehne M., Mischker A., Klinger N., Zickermann C., Walker J. (2017). Cost effects of preterm birth: A comparison of health care costs associated with early preterm, late preterm, and full-term birth in the first 3 years after birth. Eur. J. Health Econ..

[7] Garfield R.E., Maner W.L. (2007). Physiology and electrical activity of uterine contractions. Semin. Cell Dev. Biol..

[8] Leaños-Miranda A., Nolasco-Leaños A.G., Carrillo-Juárez R.I., Molina-Pérez C.J., Isordia-Salas I., Ramírez-Valenzuela K.L. (2021). Interleukin-6 in amniotic fluid: A reliable marker for adverse outcomes in women in preterm labor and intact membranes. Fetal Diagn. Ther..

[9] Devedeux D., Marque C., Mansour S., Germain G., Duchêne J. (1993). Uterine electromyography: A critical review. Am. J. Obstet. Gynecol..

[10] Fele-Žorž G., Kavšek G., Novak-Antolič Ž., Jager F. (2008). A comparison of various linear and non-linear signal processing techniques to separate uterine EMG records of term and pre-term delivery groups. Med. Biol. Eng. Comput..

[11] Garcia-Casado J., Ye-Lin Y., Prats-Boluda G., Mas-Cabo J., Alberola-Rubio J., Perales A. (2018). Electrohysterography in the diagnosis of preterm birth: A review. Physiol. Meas..

[12] Schlembach D., Maner W.L., Garfield R.E., Maul H. (2009). Monitoring the progress of pregnancy and labor using electromyography. Eur. J. Obstet. Gynecol. Reprod. Biol..

[13] Mas-Cabo J., Prats-Boluda G., Garcia-Casado J., Alberola-Rubio J., Monfort-Ortiz R., Martinez-Saez C., Perales A., Ye-Lin Y. (2020). Electrohysterogram for ANN-Based Prediction of Imminent Labor in Women with Threatened Preterm Labor Undergoing Tocolytic Therapy. Sensors.

[14] Mas-Cabo J., Ye-Lin Y., Garcia-Casado J., Díaz-Martinez A., Perales-Marin A., Monfort-Ortiz R., Roca-Prats A., López-Corral Á., Prats-Boluda G. (2020). Robust Characterization of the Uterine Myoelectrical Activity in Different Obstetric Scenarios. Entropy.

[15] Nieto-del-amor F., Beskhani R., Ye-lin Y., Garcia-casado J., Diaz-martinez A. (2021). Assessment of Dispersion and Bubble Entropy Measures for Enhancing Preterm Birth Prediction Based on Electrohysterographic Signals. Sensors.

[16] Lemancewicz A., Borowska M., Kuć P., Jasińska E., Laudański P., Laudański T., Oczeretko E. (2016). Early diagnosis of threatened premature labor by electrohysterographic recordings—The use of digital signal processing. Biocybern. Biomed. Eng..

[17] Vrhovec J., Macek A. (2012). An Uterine Electromyographic Activity as a Measure of Labor Progression. Applications of EMG in Clinical and Sports Medicine.

[18] Hassan M., Terrien J., Marque C., Karlsson B. (2011). Comparison between approximate entropy, correntropy and time reversibility: Application to uterine electromyogram signals. Med. Eng. Phys..

[19] Nieto-del-Amor F., Prats-Boluda G., Martinez-De-Juan J.L., Diaz-Martinez A., Monfort-Ortiz R., Diago-Almela V.J., Ye-Lin Y. (2021). Optimized Feature Subset Selection Using Genetic Algorithm for Preterm Labor Prediction Based on Electrohysterography. Sensors.

[20] Mas-Cabo J., Prats-Boluda G., Garcia-Casado J., Alberola-Rubio J., Perales A., Ye-Lin Y. (2019). Design and Assessment of a Robust and Generalizable ANN-Based Classifier for the Prediction of Premature Birth by means of Multichannel Electrohysterographic Records. J. Sens..

[21] Włodarczyk T., Płotka S., Szczepański T., Rokita P., Sochacki-Wójcicka N., Wójcicki J., Lipa M., Trzciński T. (2021). Machine learning methods for preterm birth prediction: A review. Electronics.

[22] Murphy K.P. (2012). Machine Learning: A Probabilistic Perspective.

[23] Carvalho D.V., Pereira E.M., Cardoso J.S. (2019). Machine learning interpretability: A survey on methods and metrics. Electronics.

[24] Sun Y., Wong A.K.C., Kamel M.S. (2009). Classification of imbalanced data: A review. Int. J. Pattern Recognit. Artif. Intell..

[25] Denil M., Trappenberg T. Overlap versus imbalance. Proceedings of the Canadian Conference on Artificial Intelligence.

[26] Vandewiele G., Dehaene I., Kovács G., Sterckx L., Janssens O., Ongenae F., De Backere F., De Turck F., Roelens K., Decruyenaere J. (2021). Overly optimistic prediction results on imbalanced data: A case study of flaws and benefits when applying over-sampling. Artif. Intell. Med..

[27] Vluymans S. (2019). Learning from imbalanced data. Stud. Comput. Intell..

[28] Chawla N.V., Bowyer K.W., Hall L.O., Kegelmeyer W.P. (2002). SMOTE: Synthetic minority over-sampling technique. J. Artif. Intell. Res..

[29] Fergus P., Idowu I., Hussain A., Dobbins C. (2016). Advanced artificial neural network classification for detecting preterm births using EHG records. Neurocomputing.

[30] Smrdel A., Jager F. (2015). Separating sets of term and pre-term uterine EMG records. Physiol. Meas..

[31] Fergus P., Cheung P., Hussain A., Al-Jumeily D., Dobbins C., Iram S. (2013). Prediction of Preterm Deliveries from EHG Signals Using Machine Learning. PLoS ONE.

[32] Ren P., Yao S., Li J., Valdes-Sosa P.A., Kendrick K.M. (2015). Improved Prediction of Preterm Delivery Using Empirical Mode Decomposition Analysis of Uterine Electromyography Signals. PLoS ONE.

[33] Koziarski M., Woźniak M., Krawczyk B. (2020). Combined Cleaning and Resampling algorithm for multi-class imbalanced data with label noise. Knowl.-Based Syst..

[34] Loyola-González O., Martínez-Trinidad J.F., Carrasco-Ochoa J.A., García-Borroto M. (2016). Study of the impact of resampling methods for contrast pattern based classifiers in imbalanced databases. Neurocomputing.

[35] Liu Y., An A., Huang X. Boosting prediction accuracy on imbalanced datasets with SVM ensembles. Proceedings of the Pacific-Asia Conference on Knowledge Discovery and Data Mining.

[36] Junsomboon N., Phienthrakul T. Combining over-sampling and under-sampling techniques for imbalance dataset. Proceedings of the 9th International Conference on Machine Learning and Computing.

[37] Park S., Park H. (2021). Combined oversampling and undersampling method based on slow-start algorithm for imbalanced network traffic. Computing.

[38] Fujiwara K., Huang Y., Hori K., Nishioji K., Kobayashi M., Kamaguchi M., Kano M. (2020). Over- and Under-sampling Approach for Extremely Imbalanced and Small Minority Data Problem in Health Record Analysis. Front. Public Health.

[39] Napierala K., Stefanowski J. (2016). Types of minority class examples and their influence on learning classifiers from imbalanced data. J. Intell. Inf. Syst..

[40] Zhou L. (2013). Performance of corporate bankruptcy prediction models on imbalanced dataset: The effect of sampling methods. Knowl.-Based Syst..

[41] Bekkar M., Alitouche T.A. (2013). Imbalanced Data Learning Approaches Review. Int. J. Data Min. Knowl. Manag. Process.

[42] Yijing L., Haixiang G., Xiao L., Yanan L., Jinling L. (2016). Adapted ensemble classification algorithm based on multiple classifier system and feature selection for classifying multi-class imbalanced data. Knowl.-Based Syst..

[43] Cunningham J.P., Ghahramani Z. (2015). Linear dimensionality reduction: Survey, insights, and generalizations. J. Mach. Learn. Res..

[44] Fu G., Xu F., Zhang B., Yi L. (2017). Chemometrics and Intelligent Laboratory Systems Stable variable selection of class-imbalanced data with precision-recall criterion. Chemom. Intell. Lab. Syst..

[45] Ramos-Pérez I., Arnaiz-González Á., Rodríguez J.J., García-Osorio C. (2022). When is resampling beneficial for feature selection with imbalanced wide data?. Expert Syst. Appl..

[46] Jager F., Libenšek S., Geršak K. (2018). Characterization and automatic classification of preterm and term uterine records. PLoS ONE.

[47] Mas-Cabo J., Prats-Boluda G., Perales A., Garcia-Casado J., Alberola-Rubio J., Ye-Lin Y. (2019). Uterine electromyography for discrimination of labor imminence in women with threatened preterm labor under tocolytic treatment. Med. Biol. Eng. Comput..

[48] Laurikkala J. Improving identification of difficult small classes by balancing class distribution. Proceedings of the Conference on Artificial Intelligence in Medicine in Europe.

[49] Babatunde O., Armstrong L., Leng J., Diepeveen D. (2014). A Genetic Algorithm-Based Feature Selection. Int. J. Electron. Commun. Comput. Eng..

[50] Nguyen M.H. (2019). Impacts of unbalanced test data on the evaluation of classification methods. Int. J. Adv. Comput. Sci. Appl..

[51] D’Agostino R.B. (1971). An omnibus test of normality for moderate and large size samples. Biometrika.

[52] Wang L., Han M., Li X., Zhang N., Cheng H. (2021). Review of classification methods on unbalanced data sets. IEEE Access.

[53] Bin Heyat M.B., Akhtar F., Abbas S.J., Al-Sarem M., Alqarafi A., Stalin A., Abbasi R., Muaad A.Y., Lai D., Wu K. (2022). Wearable Flexible Electronics Based Cardiac Electrode for Researcher Mental Stress Detection System Using Machine Learning Models on Single Lead Electrocardiogram Signal. Biosensors.

[54] Santos M.S., Soares J.P., Abreu P.H., Araujo H., Santos J. (2018). Cross-validation for imbalanced datasets: Avoiding overoptimistic and overfitting approaches. IEEE Comput. Intell. Mag..

[55] Maldonado S., Weber R., Famili F. (2014). Feature selection for high-dimensional class-imbalanced data sets using Support Vector Machines. Inf. Sci..

[56] Lin W.J., Chen J.J. (2013). Class-imbalanced classifiers for high-dimensional data. Brief. Bioinform..

[57] Vuttipittayamongkol P., Elyan E. Overlap-Based Undersampling Method for Classification of Imbalanced Medical Datasets. Proceedings of the IFIP International Conference on Artificial Intelligence Applications and Innovations.

[58] Alizadehsani R., Roshanzamir M., Hussain S., Khosravi A., Koohestani A., Zangooei M.H., Abdar M., Beykikhoshk A., Shoeibi A., Zare A. (2021). Handling of uncertainty in medical data using machine learning and probability theory techniques: A review of 30 years (1991–2020). Ann. Oper. Res..

[59] Martín-Félez R., Mollineda R.A. On the suitability of combining feature selection and resampling to manage data complexity. Proceedings of the Conference of the Spanish Association for Artificial Intelligence.

[60] Huang M.W., Chiu C.H., Tsai C.F., Lin W.C. (2021). On combining feature selection and over-sampling techniques for breast cancer prediction. Appl. Sci..

[61] Jain A., Ratnoo S., Kumar D. Addressing class imbalance problem in medical diagnosis: A genetic algorithm approach. Proceedings of the 2017 International Conference on Information, Communication, Instrumentation and Control (ICICIC).

[62] Jeni L.A., Cohn J.F., De La Torre F. Facing imbalanced data—Recommendations for the use of performance metrics. Proceedings of the 2013 Humaine Association Conference on Affective Computing and Intelligent Interaction.

[63] Japkowicz N. (2013). Assessment metrics for imbalanced learning. Imbalanced Learning: Foundations, Algorithms, and Applications.

[64] Sofaer H.R., Hoeting J.A., Jarnevich C.S. (2019). The area under the precision-recall curve as a performance metric for rare binary events. Methods Ecol. Evol..

[65] Saito T., Rehmsmeier M. (2015). The precision-recall plot is more informative than the ROC plot when evaluating binary classifiers on imbalanced datasets. PLoS ONE.

[66] Yuan Y., Su W., Zhu M. (2015). Threshold-Free Measures for Assessing the Performance of Medical Screening Tests. Front. Public Health.

[67] Acharya U.R., Sudarshan V.K., Rong S.Q., Tan Z., Lim C.M., Koh J.E., Nayak S., Bhandary S.V. (2017). Automated detection of premature delivery using empirical mode and wavelet packet decomposition techniques with uterine electromyogram signals. Comput. Biol. Med..

[68] Vuttipittayamongkol P., Elyan E., Petrovski A. (2021). On the class overlap problem in imbalanced data classification. Knowl.-Based Syst..

[69] Japkowicz N. Class imbalances: Are we focusing on the right issue. Proceedings of the Workshop on Learning from Imbalanced Data Sets II.

[70] Jo T., Japkowicz N. (2004). Class imbalances versus small disjuncts. ACM Sigkdd Explor. Newsl..

[71] Serdar C.C., Cihan M., Yücel D., Serdar M.A. (2021). Sample size, power and effect size revisited: Simplified and practical approachin pre-clinical, clinical and laboratory studies. Biochem. Med..

[72] Berghella V., Hayes E., Visintine J., Baxter J.K. (2008). Fetal fibronectin testing for reducing the risk of preterm birth. Cochrane Database Syst. Rev..

[73] Pandey M., Chauhan M., Awasthi S. (2017). Interplay of cytokines in preterm birth. Indian J. Med. Res..

[74] Sean Esplin M., Elovitz M.A., Iams J.D., Parker C.B., Wapner R.J., Grobman W.A., Simhan H.N., Wing D.A., Haas D.M., Silver R.M. (2017). Predictive accuracy of serial transvaginal cervical lengths and quantitative vaginal fetal fibronectin levels for spontaneous preterm birth among nulliparous women. Obstet. Gynecol. Surv..

[75] Lucovnik M., Chambliss L.R., Garfield R.E. (2013). Costs of unnecessary admissions and treatments for “threatened preterm labor”. Am. J. Obstet. Gynecol..

